# Sex Differences and Autism: Brain Function during Verbal Fluency and Mental Rotation

**DOI:** 10.1371/journal.pone.0038355

**Published:** 2012-06-12

**Authors:** Felix D. C. C. Beacher, Eugenia Radulescu, Ludovico Minati, Simon Baron-Cohen, Michael V. Lombardo, Meng-Chuan Lai, Anne Walker, Dawn Howard, Marcus A. Gray, Neil A. Harrison, Hugo D. Critchley

**Affiliations:** 1 Psychiatry, Brighton & Sussex Medical School, Brighton, United Kingdom; 2 Sackler Centre for Consciousness Science, University of Sussex, Brighton, United Kingdom; 3 Department of Biomedical Engineering, Stony Brook University School of Medicine, Stony Brook, New York, United States of America; 4 Scientific Department and Neuroradiology Unit, Fondazione IRCCS Istituto Neurologico “Carlo Besta”, Milano, Italy; 5 Autism Research Centre, Department of Psychiatry, University of Cambridge, Cambridge, United Kingdom; 6 Neurobehavioural Clinic, Sussex Partnership NHS Foundation Trust, c/o Sussex Education Centre, Mill View Hospital Site, Hove, United Kingdom; 7 Experimental Neuropsychology Research Unit School of Psychology, Psychiatry Faculty of Medicine Nursing & Health Sciences, Monash University Clayton, Victoria, Australia; Royal Holloway, University of London, United Kingdom

## Abstract

Autism spectrum conditions (ASC) affect more males than females. This suggests that the neurobiology of autism: 1) may overlap with mechanisms underlying typical sex-differentiation or 2) alternately reflect sex-specificity in how autism is expressed in males and females. Here we used functional magnetic resonance imaging (fMRI) to test these alternate hypotheses. Fifteen men and fourteen women with Asperger syndrome (AS), and sixteen typically developing men and sixteen typically developing women underwent fMRI during performance of mental rotation and verbal fluency tasks. All groups performed the tasks equally well. On the verbal fluency task, despite equivalent task-performance, both males and females with AS showed enhanced activation of left occipitoparietal and inferior prefrontal activity compared to controls. During mental rotation, there was a significant diagnosis-by-sex interaction across occipital, temporal, parietal, middle frontal regions, with greater activation in AS males and typical females compared to AS females and typical males. These findings suggest a complex relationship between autism and sex that is differentially expressed in verbal and visuospatial domains.

## Introduction

Autism Spectrum Conditions (ASC) are characterized by developmental impairments in communication, social and emotional functioning, alongside restricted, stereotyped or repetitive behaviors. Individuals with Asperger syndrome (AS; an increasingly recognized type of ASC) have broadly intact verbal language development though speech production may still be atypical in terms of prosody, pragmatics, rate, volume and frequency [1]. They express pervasive difficulties in social-communication and have restricted and stereotyped behaviors. Socially, AS individuals are characterized by interpersonal awkwardness and on formal psychometry they tend to display relative strengths in verbal skills and rote learning skills, but weaker visuomotor and conceptual learning abilities [1].

ASC, and in particular AS, is more commonly diagnosed in males than females. This observation, coupled with widely documented sex differences in brain structure, function and neurotransmission in the normative population [2] and in various pathological conditions (including autism) [3], have motivated research into a possible relationship between the mechanisms underlying sexual differentiation and autism. Two related theories are of immediate relevance to this issue. First is the ‘Empathizing-Systemizing’ (E–S) theory, which draws on the distinction between the cognitive domains of empathy (the drive to identify another person’s mental states and respond with an appropriate emotion) and ‘systemizing’ (the drive to analyze and construct rule-based systems) [4]. Sex differences in the general population are apparent in both these domains. For example, females on average show higher levels of empathy compared to systemizing, while males typically show the opposite profile [4]. The second, known as the ‘extreme male brain (EMB) theory’ extends the E–S theory to autism [5], [6]. Based initially on the observation of exaggerated male-typical empathizing/systemizing patterns in autism it has subsequently led to the proposal that some of the mechanisms causing autism may be linked to those related to typical sex differences (e.g., endogenous sex steroids).

To date, evidence at the behavioral, cognitive and psychometric levels has supported the EMB theory [7]. However, the EMB theory also predicts an exaggeration of typical sexual dimorphism at the brain/neural level in autism. Interestingly, a recent structural MRI study [8] instead described attenuated sex differences in males and females with AS in brain regions that typically show sex differences. A follow up study of the same dataset also reported reduced male-female differences in the textural consistency of whole brain grey matter in AS [9]. At first glance, these findings appear counter to predictions of the EMB theory, however they may also be explained by sex-specificity in autism [10], i.e. mechanisms underlying autism may be differentially expressed in males and females. There is some parallel support for this alternate view. For example, in a study of serum biomarkers, distinct sets of molecules predicted the diagnosis of AS in males compared to females (with free testosterone belonging to the set of predictors for females) [11], [12].

In the present study, we test for the first time the following hypotheses:

1. There is sex-specificity in brain function in autism.
2. This sex-specificity is expressed either qualitatively as an atypical pattern relative to the normative population, or quantitatively as an exaggeration of typical sexual dimorphism (as predicted by the EMB theory of autism).

To test these hypotheses, we scanned AS and typical individuals while they performed verbal fluency and mental rotation tasks. These tasks have been shown to be sensitive to sex and diagnosis in both behavioral and neuroimaging studies with women tending to perform better than men in verbal fluency [13] and men better than women in mental rotation [14], [15], [16], [17]. fMRI studies in autism using these types of tasks also elicit group-differences in activation [18], [19]. The selected tasks assess circumscribed cognitive domains and a direct relationship with the E–S theory may not be obvious. Nevertheless, a relative advantage of females in language domains is consistent with greater communication and empathizing abilities in females [20]. Likewise, mental rotation, where males have a relative advantage compared with females, is shown to correlate with systemizing [21].

Past work suggests that neuroimaging studies on sex differences in verbal and spatial domains should ideally control for confounding factors [22], particularly task-performance. For example, in verbal fluency tasks, studies find few differences in brain activation between men and women after controlling for task-performance [23], [24]. However, larger studies show greater recruitment of prefrontal, cingulate and temporal cortical regions in men compared to women, irrespective of performance, and greater engagement of prefrontal and cingulate cortices in men with increasing performance [25]. For mental rotation, men are frequently reported to show greater activation in right and/or bilateral parietal regions than women, while women additionally recruit more right frontal cortex [26], [27], [28]. However, when controlling for performance, sex differences diminish [23] or become exaggerated [29]. The latter may reflect adoption of distinct strategies by the two sexes during particular types of mental rotation task. In the current study, we took care to ensure that task-performance was similar across groups. This makes interpretation of group differences in activation independent of simple behavioral effects, corresponding more directly to differences in neural processing that may reflect recruitment of cognitive strategies or ancillary neural substrates to perform the task.

In the present study, we examined individuals with AS and typical controls matched on age, sex and performance during fMRI experiments. We used the verbal fluency and mental rotation tasks within factorial experimental designs to identify diagnosis, and sex specific effects as well as diagnosis-by-sex interaction effects on brain function. In addition, we used planned comparisons to identify if exaggerated typical sexual dimorphism in brain function characterizes AS individuals. To our knowledge, this is the first study investigating sex and diagnosis effects on brain functioning in AS, compared with a non-clinical population.

## Methods

### Participants

Sixty-one individuals were recruited to the study; fifteen males and fourteen females with Asperger Syndrome (14 and 13 right-handed respectively) and sixteen male and sixteen female controls (15 and 14 right-handed respectively). All participants with Asperger syndrome were diagnosed following specialist assessment in the Sussex Adult Neurobehavioural Clinic, Brighton, UK. All diagnoses were made following multidisciplinary assessment by a neuropsychiatrist (HDC & NAH), a clinical psychologist (DH) and a speech and language therapist (AW) trained in the assessment of adults with neurobehavioral disorders according to DSM-IV-TR criteria. Clinical diagnosis was validated using the Diagnostic Interview for Social and Communication Disorders (DISCO) [30]. Individuals with a history of epilepsy, neurological abnormalities, general learning disability, known history of significant head injury, or psychosis were excluded. Written informed consent was obtained in accordance with the Declaration of Helsinki (1991), and the procedures were approved by a National Health Service Research Ethics (NRES) Committee.

### Psychological Assessments

All participants completed the Autism Spectrum Quotient (AQ) [31] and the Empathy Quotient (EQ) [6]. The AQ is a measure of autistic traits in adults and has good reliability and validity that has been replicated cross-culturally. It is independent of IQ, age, education, and major personality traits [32]. The Empathy Quotient is a validated measure of empathy [33]. Additionally we used the National Adult Reading Test (NART) [34] as an index of intellectual function. All questionnaires were completed on the day of scanning. Demographic and neuropsychological scores were analyzed using two-way ANOVAs (under the General Linear Model implemented in SPSS 17.0) with sex and diagnosis as fixed factors.

### fMRI Data Acquisition

Whole brain fMRI data was acquired on a 1.5 T Siemens Magnetom Avanto magnetic resonance scanner equipped with a 12 channel head coil (Siemens Medical Systems AG, Erlangen, Germany). Functional images were acquired with a gradient echo-planar T2* sequence sensitive to blood oxygen level dependent (BOLD) contrast, each comprising a full brain volume (5 mm slice thickness, 0% gap, 50 ms echo time, 2.4 s repetition time per volume, 25 slices). 145 volumes per subject were acquired for the verbal fluency task, and 380 volumes per subject for the mental rotation task. The duration of the scanned verbal fluency task was just under six minutes and the mental rotation was just over fifteen minutes. Axial slices were tilted by 30° to reduce signal dropout in orbitofrontal cortex [35].

### Tasks

#### Verbal fluency task

The verbal fluency task was presented as a block design, composed of six 40-second task and control conditions. During each task block participants were shown one of six letters (T, L, B, O, A, N) which was displayed for the duration of the block. Their task was to think of as many words as possible beginning with the letter displayed, indicating each new word with a button press. The word generation condition was contrasted with a baseline condition, where participants repeated the series of button presses made in the previous condition. During the control condition, participants were instructed to calmly think of the word ‘REST’ while watching the computer screen. In order to match conditions for motion, participants were cued to make button presses at the same times as in the previous word generation condition by brief visual stimuli (three crosses displayed in the center of the screen). The total number of responses (button presses) across all of the task sessions was used as the outcome measure.

#### Mental rotation task

**Figure 1:**
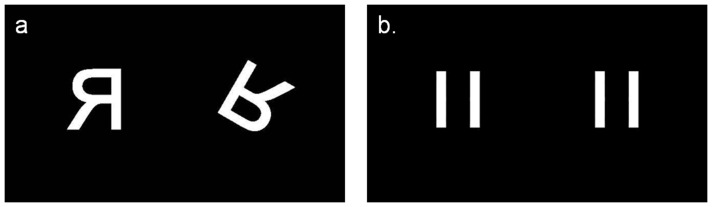
Mental rotation stimuli (a) and control condition (b).

The mental rotation task was presented and modeled as an event-related design. Participants were shown sixty pairs of white letters (F, G, J, P, and R) presented against a black background. Each pair of letters was presented pseudo-randomly (see Fig. 1a), with the right hand letter displayed at one of four different rotational angles (90, 150, 210 or 270 degrees). Letters were either matched (configurable identical - both were orthographic or mirror images) or unmatched/flipped (one letter was a mirror image of the other) (Figure 1). Participants used a button box held in the right hand to indicate if the letters were matched (index finger button press) or unmatched (middle finger button press). Letter stimuli were presented for between 8 and 12 seconds with a mean inter-stimulus interval of 10 seconds and were presented until a response was made, following which the letter was replaced with a black screen. Percentage correct responses and mean reaction times were used as outcome measures.

In the control condition, participants saw two vertical white bars presented against the same black background (see Fig. 1b) and were instructed to press either button (index or middle finger) in response to their presentation. Control stimuli were chosen to control for visual content and motor responses but had no mental rotation requirement and were presented a total of 20 times. Stimulus presentation was controlled by a program written in Cogent 2000 (http://www.vislab.ucl.ac.uk/cogent_2000.php), a toolbox of Matlab (The MathWorks Inc., Natick, Massachusetts). A Matlab program recorded response accuracy (‘correct’ and ‘incorrect’) and reaction time (ms) on each trial. A valid response could span the presentation interval (8–12 seconds).

### fMRI Processing and Statistical Analysis

Functional images were analyzed using SPM8 (Wellcome Trust Centre for Neuroimaging; http://www.fil.ion.ucl.ac.uk/spm/software/spm8/). The first four functional volumes were discarded to allow for T1 equilibration effects and the remaining volumes manually adjusted to set the origin at the anterior commissure. Images were first spatially realigned and unwarped then spatially normalized to standard MNI (Montreal Neurologic Institute) space via normalization parameters calculated from the mean functional image in a single generative model embodied in the segment routine [36]. This algorithm iteratively corrects for non-uniformities in image intensity, spatially normalizes grey matter, white matter and cerebrospinal fluid tissue classes, and segments into tissue classes. Functional scans were subsequently smoothed with an 8 mm Gaussian smoothing kernel [37].

Both tasks were modeled at the first level as event related designs, which included realignment movement parameters as covariates of no interest. In the verbal fluency task, participant button presses indicating each word generated were, due to the block-like nature of the task design, clustered together within each word generation condition. These conditions were interspersed with rest conditions that simply required button presses based on the temporal profile of responses in the previous block.

Block instructions, word generation events and rest conditions events were each modeled with unique regressors, including temporal and dispersion derivatives to account for the inter-individual variability in BOLD response in this task [38]. In the mental rotation task, the ordering of trial types was pseudo-randomly permuted to provide high estimation efficiency and modeled in an event related fashion. Rotation blocks were interspersed with control condition blocks, in which control events (timed again via button responses) were also modeled in an event related fashion. In each task, differential contrast images were estimated (task condition – control condition) for group analysis at the second level.

For each task, we conducted a second-level one-sample t-test using the above first-level contrast images. The voxel-level threshold for the main effect of the task was set at p = 0.001 uncorrected, for exploratory purpose (and as a check to ensure that the task engaged all anticipated regions). Contrast images of the main effect of task (uncorrected threshold p<0.001) across all participants were then used as an inclusive mask to constrain subsequent analyses to the set of brain regions activated during individual task performance, as previously described [39]. We then used a 2×2 ANCOVA model (fixed factors included sex and diagnosis) to identify specific effects of sex, diagnosis and diagnosis-by-sex interactions on this pattern of brain activation. The proxy measure of intelligence (NART) was included as a covariate in both analyses, as language and mental rotation ability are known to correlate with general intelligence [40]. For statistical inference at second-level analysis, we used customized software written in MATLAB [41]. This program uses the cluster extent threshold to correct for multiple comparisons. The cluster extent threshold was obtained by simulating the whole brain fMRI activation. The size of each contiguous cluster was determined in a single simulation by modeling the entire functional image matrix (64×64×25 voxels), assuming a type I error for each voxel of 0.001. After 10,000 simulations, the probability of each cluster size was determined and the cluster extent, k = 7 voxels, that is equivalent to a p<0.001 whole brain corrected significance.

Using this stringent threshold, we first tested for sex-specificity in AS by exploring main effects of diagnosis and sex, and particularly diagnosis-by-sex interactions, which formally indicate sex-specific diagnostic effects. We then used planned comparisons (applied to regions engaged by the task), to test whether AS individuals showed an exaggeration of typically sexually dimorphic activity (i.e., where typical males have greater activity than typical females), as predicted by the EMB theory. We used a linear contrast that tested for the greatest differences across groups between AS males and typical females (with AS females and typical males placed between the ‘extreme’ groups). Thus an exaggerated sexual dimorphism in AS would be supported by a pattern such as AS males > AS females ≥ typical males > typical females.

Post hoc analyses (Bonferroni test) were further performed on the extracted mean of BOLD response within the cluster of interest. Similar tests were performed to further explore interaction effects where appropriate. The mean of activation was calculated with the SPM8 utility (EasyROI; http://www.sbirc.ed.ac.uk/cyril/cp_download.html) and the analyses were performed with SPSS 17.0.

## Results

### Demographics and Behavioral Data

Participants were matched on age and sex (with no significant between-group differences in sex or age; both p>0.05; mean age AS: 32.8 yrs, SD = 9.1; mean age controls: 30.4 yrs, SD = 7.7). As anticipated, there was a significant main effect of diagnosis on AQ with a higher AQ in AS compared to controls (AS mean AQ = 36.9 (SD  = 7.05, controls mean AQ = 13.7 (SD  = 7.43), F(1,57) = 184.8; p<0.05). There was a significant main effect of sex driven by a higher AQ in females with AS compared to males with AS (females with AS mean AQ = 40.64 (SD = 4.24), males with AS mean AQ = 33.53 (SD = 7.5), F(1,57) = 11.62; p<0.05). Similarly we observed a significant main effect of diagnosis on EQ, with lower EQ in AS compared to controls (AS mean EQ = 19.45 (SD = 8.9), controls mean EQ = 45.53 (SD  = 10.35), F(1,57) = 119.08, p<0.05). There was also a significant main effect of diagnosis on NART score, people with AS having lower NART scores compared to controls (NART score average: AS = 30.3 (SD = 7.56), controls = 35.2 (SD = 6.17); F(1,57) = 7.72; p<0.01).

### Task Performance and Reaction Time

#### Verbal fluency task

There were no significant main effects of sex or diagnosis or sex-by-diagnosis interaction on the total number of responses (AS mean number of words = 55.2 (SD = 18.35), controls mean number of words = 62, SD = 19.25, F(1,57) = 1.9, n.s.); males mean number of words = 58.7, (SD = 19.73), females mean number of words = 58.8, (SD = 18.8); sex × diagnosis: F(1,57) = 0.041, n.s.).

#### Rotation task

For the mental rotation task, three females with AS performed at chance. We concluded that these participants had not correctly understood the task and subsequently excluded them from further analysis. This change did not affect matching of the groups presented above. There were no significant main effects of diagnosis or sex or sex-by-diagnosis interaction on the percentage of correct responses (AS mean percent of correct responses = 69.5 (SD = 7.5), controls mean percent of correct responses = 71.7 (SD = 5.7, F(1,54) = 1.4, n.s.); males percent of correct responses = 70 (SD = 7.3), females percent of correct responses = 71.6, (SD = 5.7), F(1,54) = 0.645, n.s.), sex × diagnosis: F(1,54) = 0.017, n.s.). Similarly, there was no significant main effect of diagnosis or sex, or interaction effect on reaction times (AS mean reaction time = 1607 ms, (SD = 542.5), controls mean reaction time = 1507 ms (SD = 476.9), F(1,54) = 0.421, n.s.); males percent reaction time = 1534.5 ms (SD = 552.67), females percent of correct responses = 1572.5 ms, (SD = 454.4), F(1,54) = 0.035, n.s.), sex × diagnosis: F(1,54) = 1.77, n.s.).

### Functional Imaging Results: Verbal Fluency Task

#### 1) Main effect of the task

Consistent with previous reports, performance of the verbal fluency task activated a matrix of brain regions that included the left inferior frontal and cingulate cortices [25].

#### 2) Results of random-effects analysis (2×2 ANOVA with NART as a covariate)

**Figure 2:**
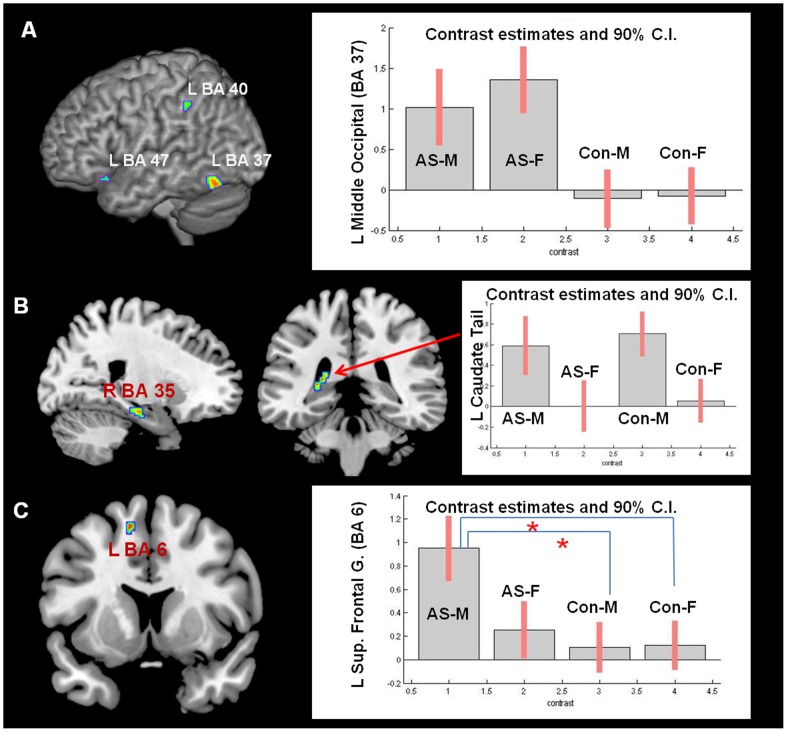
*Verbal fluency paradigm*: A. left: main effect of diagnosis (ASC > controls) within language network on the left hemisphere (middle occipital gyrus, inferior frontal gyrus, inferior parietal lobule); right: plot of effect size (parameter estimates of T contrasts) for the region showing the most statistically significant main effect of diagnosis (left middle occipital gyrus, BA 37); **B.** left and middle: main effect of sex (males> females) on left caudate and right parahippocampal gyrus; right: plot of effect size (parameter estimates of T contrasts) for the region showing the most statistically significant main effect of sex (left caudate tail). **C.** left: Cluster of activation in the left Superior Frontal gyrus showing an activity pattern based on the planned contrast: AS males>AS females ≥ typical males>typical females; **right:** plot of effect size (parameter estimates of the T contrast). Images of activation maps are thresholded at p = 0.005 uncorrected level for visualisation purpose and are overlaid on a standard template with MRICRON software (http://cnl.web.arizona.edu).

We observed a significant main effect of diagnosis, with the AS group showing greater activation compared to controls in the left middle occipital gyrus, contiguous with the fusiform word form area, and in the left inferior frontal gyrus and left inferior parietal lobule (Table 1, Figure 2A). In the typical controls, we did not find previously reported sex differences after controlling for performance [25]. However, there was a significant main effect of sex (males >females) on left caudate activity and on the right parahippocampal gyrus activity (Table 1, Figure 2B). No sex-by-diagnosis interaction was observed. In addition, there were no apparent main effects of controls > AS or females > males.

**Table 1:** Differences in brain activity during the verbal fluency task.

Region	Hemisphere	Brodmannarea (BA)	x	y	Z	Z score	Cluster extent (k)
*A. Main effect of diagnosis (AS> controls)*							
Middle Occipital G	L	BA 37	−54	−62	−14	4.58	53
Inferior Frontal G	L	BA 47	−48	22	−14	3.44	7
Inferior Parietal lobule	L	BA 40	−42	−40	44	3.38	16
*B. Main effect of sex (males > females)*							
Caudate	L	−	−24	−34	13	3.97	25
Parahippocampal G	R	BA 35	22	−16	−16	3.70	15

* L = left; R =  right; G =  gyrus.

#### 3) Post hoc analyses based on the planned linear contrast

We next tested if any of the brain regions activated by the verbal fluency task (i.e., in the mask of main effect of task across all participants) showed the pattern of activity predicted by the linear contrast AS males > AS females ≥ typical males> typical females.

The only region that showed a pattern of activity partially suggestive for exaggerated sexual dimorphism in AS was located within the left superior frontal gyrus, on the medial side (MNI: −12 10 56, Z = 3.63, k = 10, p<0.05 after FWE correction) (Figure 2C). However, post hoc analyses of this activity (mean of BOLD response extracted from the cluster in this area) revealed a significant difference between males with AS and typical males and females, but not between AS males and AS females. In fact, females with AS did not differ significantly from any other group (Table 2).

**Table 2:** Results of post hoc pair wise comparisons (Bonferroni test) showing between groups significant differences in the L Superior Frontal Gyrus activation (verbal fluency task).

	Groups	Mean BOLD gr.1∶0.55063	Mean BOLD gr.2∶0.63250	Mean BOLD gr.3∶0.80143	Mean BOLD gr.4∶1.1620
**1**	Gr1: Control females		p = 0.968326	p = 0.536615	p = 0.007804
**2**	Gr2: Control males	p = 0.968326		p = 0.800616	p = 0.026612
**3**	Gr3: AS females	p = 0.536615	p = 0.800616		p = 0.235901
**4**	Gr4: AS males	p = 0.007804	p = 0.026612	p = 0.235901	

* Legend: gr = group; p = p value; significant post hoc comparisons in red; AS =  Asperger Syndrome; L = left; mean BOLD: signal extracted from the region significantly activated for the planned contrast: **AS males>AS females ≥ typical males>typical females.**

### Functional Imaging Results: Mental Rotation Task

#### 1) Main effect of the task

The mental rotation task activated a matrix of brain regions including bilateral superior parietal, frontal and infero-temporal cortex, broadly consistent with previous reports [42].

#### 2) Results of random-effects analysis (2×2 ANOVA with NART as a covariate)

**Figure 3:**
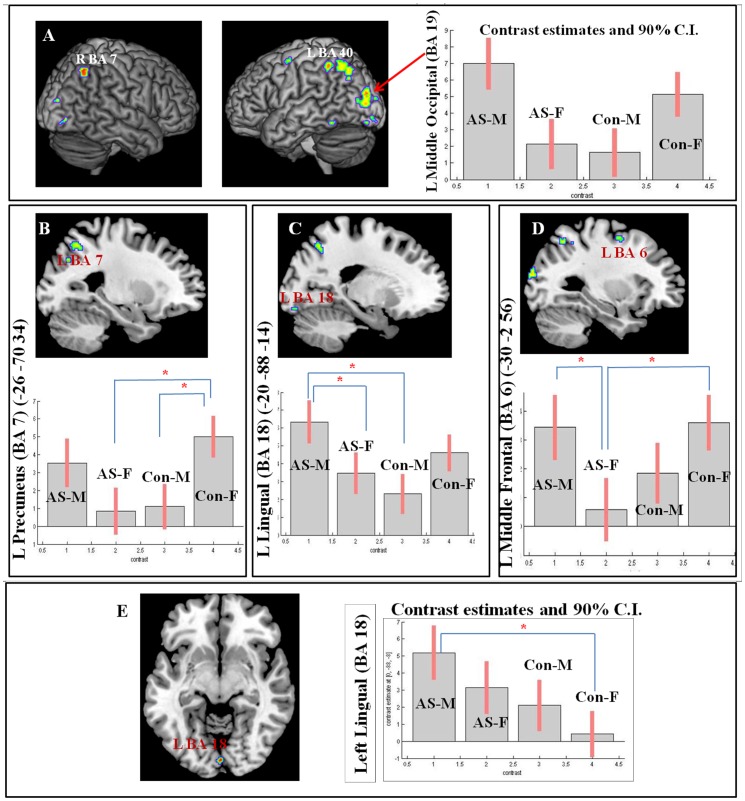
Mental rotation task: **A.** **left and middle:** interaction effect on left middle occipital gyrus (BA 19), right inferior parietal lobule (BA 7), left inferior parietal cortex (BA 40); **right:** plot of effect size (parameter estimates of the interaction T contrast) **B, C, D:** Results of post hoc analyses showing the regions and the types of effects driving an apparent interaction with the corresponding plots of effect size (parameter estimates of T contrast). **E.** Cluster of activation in the left Lingual gyrus showing an activity pattern based on the planned contrast: AS males>AS females ≥ typical males>typical females; **right:** plot of effect size (parameter estimates of the T contrast). Images of activation maps are thresholded at p = 0.005 uncorrected level for visualisation purpose and are overlaid on a standard template with MRICRON software (http://cnl.web.arizona.edu). *Legend:* L = left; R = right.

There was a significant main effect of sex (males > females) in the left cerebellum, and a sex-by-diagnosis interaction effect, comprising regions activated by the mental rotation task, (Table 3, Figure 3A). No main effect of diagnosis was observed for the mental rotation task. Several regions showed an interaction effect, with higher activity in males with AS and typical females. These included the left middle occipital gyrus, the left and right inferior parietal lobules and a cluster of extrastriate cortex extending into the middle temporal gyrus (V5/MT) (figure 3A).

**Table 3:** Differences in brain activity during the mental rotation task.

Region	Hemisphere	Brodmannarea (BA)	x	y	z	Z score	Cluster extent (k)
*A. Main effect of sex (males > females)*							
L Cerebellum (Declive)		−	−20	−80	−22	4.21	55
*B. Positive effect of interaction sex X diagnosis*							
Middle Occipital G	L	BA 19	−34	−90	16	4.34	131
	L	BA 18	−20	−88	−14	3.63	10
	R	BA 19	30	−90	16	3.33	9
	R	BA 19	46	−82	−4	3.52	9
Inferior Parietal lobule	L	BA 40	−34	−48	50	4.11	37
	R	BA 7	32	−60	48	3.78	48
Precuneus	L	BA 7	−26	−70	34	4.01	11
	L	BA 7	−22	−60	46	3.72	103
Inferior Temporal G	L	BA 20	−50	−54	−18	3.63	8
Middle Frontal G	L	BA 6	−30	−2	56	3.45	16

* L = left; R =  right; G =  gyrus.

Post hoc analyses (mean of BOLD signal extracted from significant clusters) were conducted to identify the basis of the interaction effect (Table 4 and Figure 3 B, C, D). These analyses revealed that the interaction was, in different regions, attributable to: a) hyperactivity in typical females when compared to both typical males and females with AS (Figure 3B); b) hyperactivity in males with AS when compared to both females with AS and typical males (in left precuneus, left middle occipital gyrus, left inferior temporal gyrus, right middle occipital gyrus; Figure 3C); or c) by a hypoactivity in females with AS relative to both males with AS and typical females (Figure 3D).

**Table 4:** Results of post hoc pair wise comparisons (Bonferroni test) showing the groups who trigger the interaction effect in the mental rotation task.

Region	Groups	ASC females	Control males
L Inf Parietal Lobule (BA 40)	ASC males	2.69 (0.85)*	2.37 (0.77)*
	Control females	2.49 (0.84)*	2.15 (0.76)*
R Inf Parietal Lobule (BA 7)	ASC males	3.06 (0.98)*	2.68 (0.89)*
	Control females	2.67 (0.97)*	NS
L Precuneus (BA7)	ASC males	5.1 (1.35)**	3.73 (1.23)*
	Control females	**NS**	**NS**
L Inf Temporal Gyrus (BA 20)	ASC males	2.58 (0.84)*	2.47 (0.76)*
	Control females	**NS**	**NS**
R Middle Occipital Gyrus (BA 19)−1	ASC males	2.96 (0.98)*	3.7 (0.89)**
	Control females	**NS**	**NS**
R Middle Occipital Gyrus (BA 19)−2	ASC males	4.13 (1.05)**	2.73 (0.95)*
	Control females	**NS**	**NS**

The values within cells represent: mean difference between the respective groups (i.e. ASC males > ASC females) (standard error) * or **, where *  = <0.05 and **  = <0.01; L = left; Inf = inferior; BA = Brodmann area; ASC = Autism Spectrum Conditions; NS = not significant. R Middle Occipital Gyrus 1 = MNI: 30–90 16; 2 = MNI: 46–82-4.

#### 3) Post hoc analyses based on the planned linear contrast

One region showed the predicted pattern of activity in accordance with the linear contrast AS males > AS females ≥ typical males > typical females, namely the lingual gyrus (MNI: 0 −88 −8, Z = 3.47, k = 18) (Figure 3E). Post hoc analyses of this effect (extracted mean BOLD response of cluster) showed a significant difference between males with AS and typical females only (Table 5). No significant normative sexual dimorphism was demonstrable for this area and the females with AS did not significantly differ from any other group with respect to this activation.

**Table 5:** Results of post hoc pair wise comparisons (Bonferroni test) showing between groups significant differences in the L Lingual Gyrus activation (mental rotation task).

	Groups	Mean BOLD gr.1∶0.55063	Mean BOLD gr.2∶0.63250	Mean BOLD gr.3∶0.80143	Mean BOLD gr.4∶1.1620
**1**	Gr1: Control females		p = 0.472252	p = 0.140448	p = 0.003366
**2**	Gr2: Control males	p = 0.472252		p = 0.950988	p = 0.251668
**3**	Gr3: AS females	p = 0.140448	p = 0.950988		p = 0.453012
**4**	Gr4: AS males	p = 0.003366	p = 0.251668	p = 0.453012	

* Legend: gr = group; p = p value; significant post hoc comparisons in red; AS =  Asperger Syndrome; L = left; mean BOLD: signal extracted from the region significantly activated for the planned contrast: **AS males>AS females ≥ typical males>typical females.**

## Discussion

The present study addressed similarities and differences in brain function in men and women with and without autism to show that interactions between sex and the diagnosis of AS in terms of brain function occur in a task-dependent manner. Prior work suggests that etiological and developmental mechanisms underlying autism overlap with mechanisms underlying sexual differentiation. There is also evidence that the biological and clinical expression of autism is sex-specific (i.e. different in males and females) [43], [44], [11]. The EMB theory predicts that the effects of autism (in terms of specific aspects of cognition and biology) reflect an exaggeration of sexual dimorphism observed in the typical population. We tested these predictions at the level of brain function, using verbal fluency and mental rotation tasks that are known to be sensitive to sex and diagnosis.

### Behavioral Effects

We observed that within the AS group, women self-reported higher AQ scores than men. One earlier study of AS did not find such male-female differences in AQ score [31]. However, our finding matches that of another recent study where diagnosis was formally confirmed with the Autism Diagnostic Interview-Revised (ADI-R) [44]. Confirmation of diagnosis using validated standardized instruments (i.e. DISCO, ADI-R) appears important, and differences in inclusion criteria (AS or a mixture of AS and autistic disorder [31], [44]) may also contribute to this discrepancy. Nevertheless, many high-functioning women on the autism spectrum may be under-diagnosed despite marked symptoms [43], [44], [45], [46].

For the verbal fluency and mental rotation tasks, prior work highlights the need to control for differences in task-performance when interpreting differences in activation. In this study, there were no group differences in performance or reaction time on either the mental rotation or verbal fluency tasks. This lack of a group difference in behavior is important to underscore, as it allows us to make inferences about activation differences unconfounded by performance. Our results also concur with earlier neuroimaging studies that treated performance as a confound and did not find sex differences between typical control volunteers (i.e., when considering only the non-ASC half of the participants) at a stringent significance threshold [23], [24].

### Verbal Fluency

Our neuroimaging analysis of the verbal fluency task showed a main effect of diagnosis within left middle occipital gyrus (encompassing the Word Form Area [47]), left inferior frontal gyrus and left inferior parietal lobule. This effect was driven by enhanced activation in the AS group compared to controls. Broadly, individuals with AS recruit more cortical resources during word generation. This was a general effect observed across both males and females with AS. The regions all contribute to language and the development of social communication skills. Fusiform and audiovisual association cortices are known to be affected in autism, and potentially compromise other functions such as face processing [48], [49], [19], [50]. Interestingly the cluster in fusiform cortex included the visual word form area, a center for orthographic representation, suggesting strategic recruitment by AS individuals of visual centers for word generation. It is worth noting that previous studies demonstrated sex effects only with respect to asymmetry of activation in relevant tasks [51]. This suggests that not only sex-related mechanisms operate to influence brain function in ASC.

Communication difficulties are intrinsic to ASC: while language development follows an apparently normal course in AS, subtle linguistic problems often exist, reflected in speech idiosyncrasies, pragmatic deficits, and compromised language functions such as word generation [52], [53]. It may be that the same neurodevelopmental mechanisms compromising these adjacent cortical regions also affect functions related to word representation or generation. Two other core regions for language processing were hyperactive in AS compared to controls: the left inferior frontal gyrus and the left inferior parietal lobule. Both regions are implicated as components of a larger network supporting semantic processing such as semantic retrieval [54], [55]. Left inferior frontal gyrus is also typically sensitive to semantic incongruence and ASC studies have found attenuation in responsiveness of this region to manipulations of semantic congruity [56], [57]. Left inferior frontal gyrus is also a site of convergence of cognitive and emotional information, involved in affective aspects of language processing, semantics and visual memory [58], [59]. Abnormal engagement of this region by people with AS may be linked to other perceptual and expressive deficits in affective communication.

Similarly, left inferior parietal lobule contributes to classification and comprehension in language processing [60], [54]. Previous fMRI studies in ASC demonstrate abnormal activation in the inferior parietal lobule, manifested as hypo- or hyperactivity depending on the elicited language sub-process [61], [62]. In our study, we interpret the hyperactivation in language areas in AS individuals as an implementation of less efficient neural processes for semantic retrieval and word generation operations, augmented by visual and/or orthographic representations of words. This contrasts with typical individuals who engage efficient phonological lexical strategies. This interpretation remains speculative as no behavioral differences were observed on this task.

We did not find evidence for typical sex differences on the verbal fluency task. One reason could be a general yoking of sex differences to behavioral performance. While we wanted to reduce effects of behavioral performance on activation differences, this may have inadvertently attenuated sex differences in the fMRI data. One region (left medial frontal gyrus) displayed significantly greater activity in AS males relative to controls, yet we cannot interpret this result as support for an exaggeration of typical sex differences in autism, since control males did not differ from control females in activation of this region (i.e. no typical sex differences were observed). However, over-recruitment of left medial frontal gyrus in AS males raises interesting questions in itself. Notably, this region shows hyperactivation during effortful, cognitively demanding conditions as observed in non-clinical populations during performance of a verbal fluency paradigm [63], numerical Stroop task [64], syntactical language production in bilinguals [65], or processing of conflicting information in Theory of Mind [66]. This result deserves further investigation since such functions are relevance to autism. Overall though, there was no evidence to support sex-specific effects on brain function in AS during verbal fluency, evidenced by the lack of sex-by-diagnosis interaction effects throughout the brain.

### Mental Rotation

Unlike data from the verbal fluency task, fMRI data from the mental rotation task showed no evidence of main effects of diagnosis. However, in several regions a sex-by-diagnosis interaction effect was present. The presence of sex-by-diagnosis interactions suggest sex-specificity in how the recruitment of neural systems supporting mental rotation are affected in autism. In particular, occipito-parietal-temporal areas were hyperactive in AS males compared to control males. However in females, control participants showed heightened activation compared to women with AS.

During mental rotation, the interaction between sex and diagnosis in the activity of pertinent regions seems to be complex. Previous neuroimaging studies of mental rotation tend to report enhanced right parietal lobule activity in typical men relative to women, while typical women engage more the right inferior frontal gyrus relative to typical men [26], [28]. One interpretation of these observations is that in the general population the two sexes use different strategies during mental rotation tasks, with men relying more on a ‘gestalt’ analysis, whereas women employ a more piecemeal serial analytic strategy [26]. However, this explanation for sex differences in brain functionality during mental rotation hides greater complexity: when matched for similar performance, women may show greater activity than men in parietal regions [29]. Furthermore, some studies fail to find sex differences during mental rotation, in either parietal or inferior frontal cortical regions [23]. In our study, similar performance across our participant groups may thus account for a lack of a significant main effect of sex (or a difference between typical men and women) in the activity of parietal or frontal cortices [23], [29]. Only one region, the cerebellum, reflected a main effect of sex in this task, showing greater task-induced activity in males (as observed in a previous study [67]).

Several aspects should be accounted for when examining sex differences in brain activity during mental rotation. Mental rotation involves different component processes (e.g. perceptual representation of orientation, analytic appraisal, rotational imagery, and comparative judgment). These component processes are likely supported by dissociable brain systems [68], particularly different subregions within parietal cortex. Thus, it is plausible that systematic differences in the deployment of particular subcomponents contribute to the observed sex-by-diagnosis interactions. Future research is required to detail the underlying mechanisms. With reference to our results, we speculate that the recruitment of occipito-parietal regions by AS males and typical females might reflect a less efficient strategy for task performance that emphasizes local feature processing. In males with ASC it may also reflect a primary advantage for local visual processing [69]. Evidence supporting this interpretation comes from the finding that individuals with ASC show enhanced performance on visual reasoning (e.g. Raven’s Standard Progressive Matrices) and visual search tasks, and the corresponding enhanced recruitment of parietal and extrastriate areas [70], [71]. However, these explanations do not satisfactorily account for why typical females show similar levels of recruitment as AS males.

The only brain region where mental rotation activity could be interpreted as an exaggeration of typical sexual dimorphism in the AS group was the lingual gyrus. This is a visual region associated with processing of affective and socially relevant information [72], [73], [74]. Our finding motivates further attention to this region in AS, as it again implicates temporo-parieto-occipital regions in functional expression of social-cognitive abilities [74], [75]. Nevertheless, without over-interpreting mechanisms, the main inference from the current sets of results, especially the noted sex-by-diagnosis interactions in various temporo-parieto-occipital regions, is that males and females with AS are different in how they recruit neural systems for mental rotation operations.

### Limitations

There are some limitations to the present study. First, we studied only individuals with Asperger syndrome (AS). Our results may therefore not apply to individuals with other subtypes of ASC. Second, the groups were not completely matched for overall cognitive function; the AS group scored lower on a proxy measure of general intelligence (i.e., NART). Nonetheless, each participant was in the average range of intelligence, performance on the tasks did not differ between groups, and we included NART score as a potentially confounding covariate throughout our neuroimaging analyses. Third, a language task was used, but we did not conduct a parallel comprehensive characterization of verbal abilities in the AS or control group. However, none of the participants in our study displayed overt language abnormalities or significant histories of such problems. Furthermore, a history of general learning disability was among the exclusion criteria. Finally, the sample size was relatively small for each group. Though our samples sizes conform with those typically utilized for between group analyses of fMRI data, between group differences at the behavioral level typically emerge in studies of larger populations. Similarly larger studies might detect more subtle differences in the brain, including the exaggerated typical sexual dimorphism in ASC, which might support the EMB theory.

### Conclusions

Despite these limitations, this study is the first direct examination of how sex and diagnosis may independently or interactively affect brain function in autism. During a verbal fluency task, we showed generalized diagnosis effects in ASC (irrespective of being male or female) within regions involved in word representation and semantic processing. During visuospatial processing (mental rotation), we observed several interaction effects that indicate sex-specificity in how brain function is affected in autism. Overall, the complex behavioral and imaging effects invite a flexible interpretation: for some cognitive processes (i.e. in language domains) males and females with ASC behave as a homogeneous group, whereas for others (i.e. visuospatial processing), the differential patterns of brain function hint at the validity of considering males and females as distinct sub-groups on the autism spectrum.
